# Regorafenib modulation of the angiopoietin/TIE2 axis in a mouse model of sepsis-induced lung injury

**DOI:** 10.25122/jml-2023-0135

**Published:** 2023-11

**Authors:** Mohammed Hamzah Ibadi, Sahar Majeed, Fadhaa Abdulameer Ghafil, Najah Rayish Hadi

**Affiliations:** 1 Babylon Health Directorate, Babylon, Iraq; 2 Department of Pharmacology and Therapeutics, Faculty of Medicine, University of Kufa, Najaf, Iraq

**Keywords:** CLP, sepsis, regorafenib, VEGF, cadherin, Ang/TIE2 axis, Ang: Angiopoietin, NF-κB: Nuclear Factor kappa-light-chain-enhancer of activated B cells, VEGF: Vascular Endothelial Growth Factor, IL-1β: Interleukin-1 beta, TNF-α: Tumor Necrosis Factor-alpha, qPCR: Quantitative Real-Time PCR, MPO: Myeloperoxidase, TIE2: Tyrosine Kinase with Immunoglobulin-like and EGF-like Domains 2, CLP: Cecal Ligation and Puncture, HKG: Housekeeping Gene

## Abstract

Sepsis, often resulting from an immune response overreaction to microorganisms and their products, can lead to acute lung injury through inflammation mediated by excessive cytokines. This study aimed to investigate the effects of regorafenib on lung injury in mice following the induction of sepsis. We divided mice into four groups (n=6 each): a sham group (undergoing laparotomy without cecal ligation and puncture [CLP]), a CLP group, a vehicle group, and a regorafenib-treated group (30 mg/kg IP, administered one hour before CLP). TNF-α, IL-1β, VEGF, MPO, caspase-11, and Ang-2 levels were significantly increased (p<0.05) in the CLP group compared to the sham group, while the regorafenib group showed significant reductions in these markers *versus* the CLP group (p< 0.05). In contrast, Ang-1 levels, which were reduced in the CLP group (p<0.05) compared to the sham group, were elevated in the regorafenib group compared to the CLP group. Quantitative real-time PCR revealed a significant decrease in TIE2 and VE-cadherin mRNA expression in the lung tissue of the CLP group compared to the sham group. There were no significant differences in mRNA expression of the TIE2 gene between the regorafenib and CLP group. However, VE-cadherin significantly increased after regorafenib treatment. Regorafenib demonstrated lung-protective effects through its anti-inflammatory and antiangiogenic activities and its influence on lung tissue mRNA expression of the cadherin gene.

## INTRODUCTION

Sepsis represents a life-threatening condition where the body's response to an infection becomes abnormal, leading to organ dysfunction. This condition is marked by a concurrent state of unbalanced immune suppression and hyperinflammation [1]. One of the key mechanisms of sepsis-induced damage is vascular leakage, resulting from endothelial dysfunction, which significantly contributes to the high mortality rates associated with this condition. Disturbance of the angiopoietin/tyrosine kinase with immunoglobulin-like and VEGF-like domains 2 (Ang/Tyrosine Kinase with Immunoglobulin-like and EGF-like Domains (TIE2) pathways may cause endothelial activation and lead to sepsis [2].

Regorafenib is a cytotoxic drug and a multi-kinase inhibitor known to target receptors such as TIE2 and vascular endothelial growth factor receptors 1-3 (VEGFR 1-3). Regorafenib significantly inhibits TIE2 and vascular endothelial growth factor (VEGF) receptors, which can significantly reduce vascularization [3]. There is a current gap in the knowledge regarding the potential for regorafenib, known primarily as a cytotoxic drug, to be utilized in treating lung sepsis by enhancing endothelial integrity via its effects on the Ang/TIE2 pathway. This study aimed to investigate the protective effects of regorafenib on lung injury in a mouse model of sepsis, examining its impact on endothelial function and inflammatory markers.

## MATERIAL AND METHODS

### Study location and design

This study was conducted at the Department of Pharmacology and Therapeutics at the Faculty of Medicine, University of Kufa. Twenty-four male Swiss albino mice, aged 8-12 weeks and weighing 25-30 g, were obtained from the College of Science, University of Kufa. Mice were housed under a 12:12 light-dark cycle at a stable temperature of 25°C and 60-65% humidity, with *ad libitum* access to water. The animals were allowed a 14-day acclimatization period in the housing facility before the start of the experiment. Following this, mice were allocated into four groups with six animals each: a sham group, which underwent a laparotomy without cecal ligation and puncture (CLP); a CLP group, which underwent the CLP procedure; a vehicle group, which received an intraperitoneal (IP) injection of a 10% solution of dimethyl sulfoxide (DMSO) one hour before CLP; and a regorafenib-treated group, which received 30 mg/kg of regorafenib IP one hour before CLP. All animals were euthanized 24 hours post-CLP. regorafenib was observed to significantly reduce macrophage infiltration and demonstrated a strong antiangiogenic effect at this dosage.

### Experimental procedure

Polymicrobial sepsis was induced in a selected group of mice based on previous studies. A double-puncture technique using 20-gauge needles was used to induce polymicrobial sepsis. Mice were anesthetized with a 1:1 solution of ketamine (75 mg/kg) and xylazine (15 mg/kg) administered intraperitoneally. A 1.5-cm midline incision was made to expose the cecum just below the ileocecal valve, which was then ligated and punctured. A small amount of fecal matter was extruded to confirm patency. The cecum was anatomically repositioned, and the abdominal incision was closed with a 5.0 surgical suture. Post-surgery, mice received 20 mL/kg of normal saline for resuscitation. Symptoms such as lethargy, fever, piloerection, diarrhea, huddling, and malaise were monitored as indicators of sepsis [4].

### Regorafenib preparation

Regorafenib powder, supplied by Macklin Inc., was dissolved in 10% DMSO and administered intraperitoneally at a dose of 30 mg/kg one hour before the CLP procedure [5].

### Collection of lung tissue samples

Mice were euthanized with a ketamine-xylazine anesthetic 24 hours following CLP. Lung tissues were harvested and divided into three parts: one for homogenization and enzyme-linked immunosorbent assays (ELISA), another preserved in RNA solution for subsequent quantitative real-time polymerase chain reaction (qRT-PCR) analysis, and the last part fixed in formalin for histopathological examination.

### Lung tissue homogenization

The lung tissue homogenization technique was carried out in accordance with methodologies established by Aziz and colleagues [6].

### Tissue preparation for histopathology

Histopathological examination and scoring of the lung tissues were conducted using the Mikawa method [7].

### Expression of TIE2 and VE-cadherin in lung tissue

The mRNA expression levels of the TIE2 and vascular endothelial (VE)-cadherin genes were quantified using qRT-PCR, following the manufacturer's instructions. Total RNA extraction was performed using specialized reagents and equipment, as described in a previous study [8].

### Primer preparation

The primer sequences used for the gene expression quantification of TIE2, VE-cadherin, and the housekeeping gene (HKG) are detailed in Table 1.

**Table 1:** Primer sequences used in gene expression quantification of TIE2, VE-cadherin, and HKG

GENE	F	R
TIE2	5’-GCCGCGGACTGACTACGAGC-3’	5’-GGAGGAGGGAGTCCGATAGACGC-3’
HKG	TGGCCTTCCGTGTTCCTAC	GAGTTGCTGTTGAAGTCGCA
VE-cad	GCAATGGCAGGCCCTA ACTTTC	CAGCAAACTCTCCTTGGA GCAC
HKG	TGGCCTTCCGTGTTCCTAC	GAGTTGCTGTTGAAGTCGCA

### Statistical analysis

We used the Statistical Package for the Social Sciences (SPSS) software, version 26 for statistical analysis. The students' t-test and one-way analysis of variance (ANOVA) with least significant difference (LSD) post hoc tests were employed to assess differences between groups. Statistical significance was established at p-value ≤ 0.05.

## RESULTS

### Effect of regorafenib on tissue levels of inflammatory and angiogenic markers

**Figure 1:**
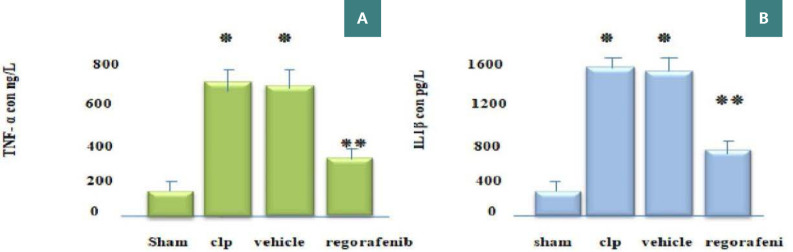
Mean tissue levels of (A) TNF-α (ng/L) and (B) IL-1β (pg/L) in the experimental groups. *p: Significant difference p<0.05 compared to the sham group. **p: Significant difference p<0.05 compared to the CLP group.

**Figure 2:**
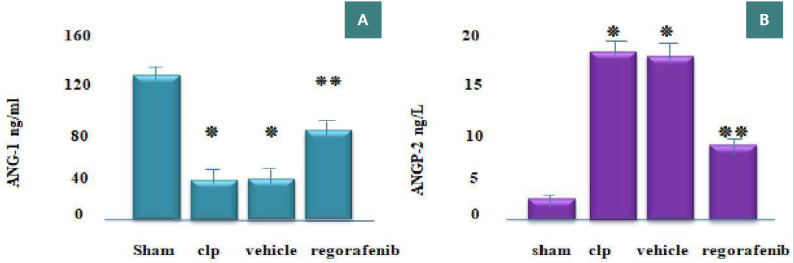
Mean tissue levels of (A) Ang-1(ng/mL) and (B) Ang-2 (ng/mL) in the experimental groups. *p: Significant daifference p<0.05 compared to the sham group. **p: Significant difference p<0.05 compared to the CLP group.

**Figure 3:**
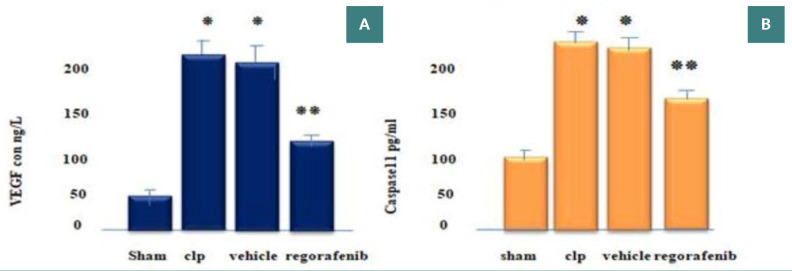
Mean tissue level of (A) VEGF (ng/L) and (B) caspase-11 (pg/mL) in the experimental groups. *p: Significant difference p<0.05 compared to the sham group. **p: Significant difference p<0.05 compared to the CLP group.

**Figure 4:**
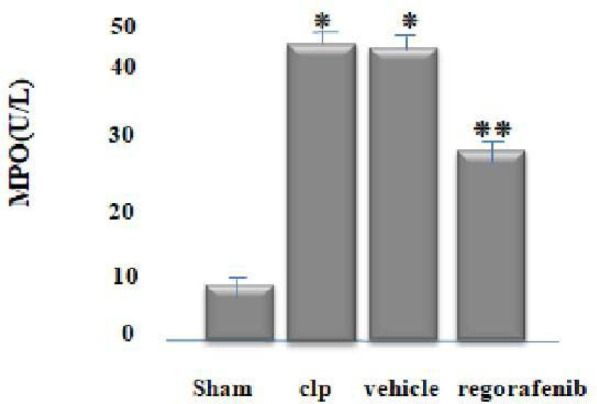
Mean tissue level of MPO (U/L) in the experimental groups. *p: Significant difference p<0.05 compared to the sham group. **p: Significant difference p<0.05 compared to the CLP group.

There was a significant increase in tissue levels of tumor necrosis factor-alpha (TNF-α), interleukin-1 beta (IL-1β), myeloperoxidase (MPO), angiopoietin-2 (Ang-2), vascular endothelial growth factor (VEGF), and caspase-11 in the CLP and vehicle groups compared to the sham group (p<0.05). Conversely, the regorafenib-treated group had significantly lower levels of these markers compared to the sepsis-induced (CLP) and vehicle groups (p<0.05). Furthermore, the level of Ang-1 was significantly lower in the CLP and vehicle groups compared to the sham group, while the regorafenib group had significantly higher levels of Ang-1 compared to CLP and vehicle groups (p<0.05) (Figures 1-4).

### Effect of regorafenib on mRNA expression of TIE2 and VE-cadherin

**Figure 5:**
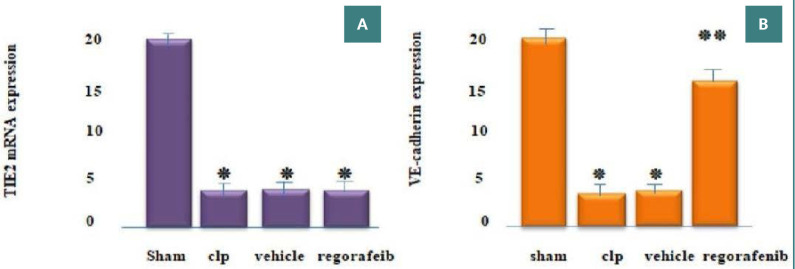
Mean tissue level of (A) TIE2 and (B) VE-cadherin mRNA expression in the experimental groups. *p: Significant difference p<0.05 compared to the sham group. **p: Significant difference p<0.05 compared to the CLP group.

Quantitative real-time PCR analysis showed a significant decrease in the mRNA expression of the TIE2 and VE-cadherin genes in the sepsis (CLP) and vehicle groups compared to the sham group (p<0.05). While there was no significant difference in the mRNA expression of the TIE2 gene between the regorafenib and CLP groups, there was a significant decrease when compared with the sham group (clinically insignificant) (p<0.05) (Figure 5A). VE-cadherin expression was significantly higher in the regorafenib group than in the CLP and vehicle groups, as demonstrated in Figure 5B.

### Effect of regorafenib on lung histopathology

**Figure 6:**
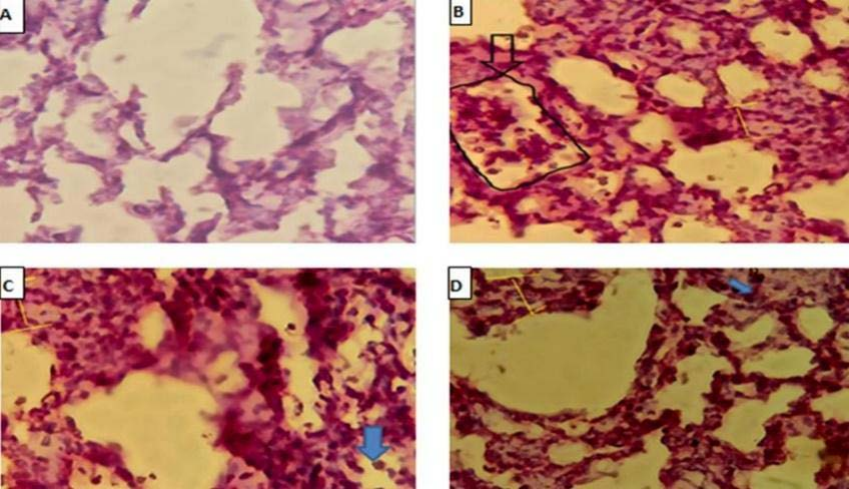
Histopathological examination of lung tissue (H&E, 400x). (A) Sham group: Normal lung histology with clear cellular borders and structures, Grade 0. (B-C) CLP and vehicle group: neutrophilic infiltration (blue arrow), alveolar wall thickening (yellow arrow), and focal areas of congestion (black arrow), Grade 4. (D) regorafenib group: Mild neutrophilic infiltration (blue arrow) and focal alveolar wall thickening (yellow arrow).

The histological examination in the sham group showed normal lung tissue. In contrast, the CLP and vehicle group showed significant lung damage (p<0.05) compared to the sham group, characterized by alveolar congestion, hemorrhage, neutrophil infiltration, and thickening of the alveolar wall, with a highly severe histological score of 4/4. On the other hand, the regorafenib group demonstrated a significant reduction in lung injury (p<0.05). The extent of changes in this group ranged from negligible to moderate, resulting in a histological grade of 2/4 (Figure 6).

**Figure 7:**
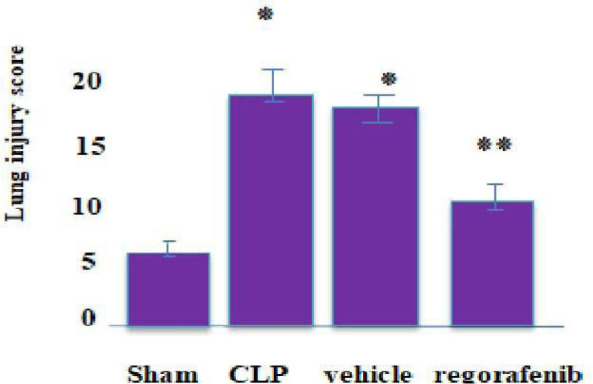
Difference in lung damage mean rank scores. *p: Significant difference p<0.05 compared to the sham group. **p: Significant difference p<0.05 compared to the CLP group.

The lung injury score was assessed using the Mikawa method [7], which grades lung injury from 0 to 4 across four categories: alveolar congestion, hemorrhagic areas, neutrophilic infiltration, and alveolar wall thickening. The total lung injury score was calculated by summing the scores from each category (Figure 7).

## DISCUSSION

This study revealed that TNF-α and IL- 1β levels were significantly elevated in the CLP and vehicle groups compared to the sham group, compatible with the results of another study [9]. This increase in the sepsis group is likely attributed to the activation of macrophages, including liver Kupffer cells, which produce various inflammatory mediators such as IL-1β [10]. Furthermore, within our study, tissue levels of TNF-α were significantly reduced in the regorafenib group compared to the CLP and vehicle groups. This finding is consistent with Han *et al*., who demonstrated that regorafenib significantly mitigated lipopolysaccharide (LPS)-induced secretion of IL-1β and TNF-α in a mouse model of Alzheimer's disease, suggesting a regulatory effect on the inflammatory response [11]. The decreased tissue levels of TNF-α and IL-1β in the regorafenib group may be attributed to its suppressive action on the NF-κB, which induces the expression of different pro-inflammatory genes, including those encoding cytokines and chemokines such as TNF-α and IL-1β, and inflammasome activity [12].

Furthermore, our study showed that Ang-1 tissue levels were significantly lower in the CLP and vehicle groups compared to the sham group. This aligns with the findings of Stenzel *et al*., which suggest that high VEGF levels correlate with increased endothelial permeability, severity of illness, and mortality in sepsis. Conversely, Ang-1 contributes to endothelial stabilization, opposing the effects of VEGF [13]. The decline in Ang-1 may be linked to sepsis-induced vascular destabilization and proangiogenic effects, which result in increased permeability and organ failure due to TIE2 antagonism by Ang-2 [14]. The regorafenib group showed a significant increase in tissue levels of Ang-1 compared to the CLP group, which, to our knowledge, is a novel finding in the context of the CLP model of sepsis in mice. This increase could be due to the inhibitory effects of regorafenib on Ang-2, which antagonizes Ang-1, or its enhancement of hypoxia-inducible factor 2 alpha (HIF-2α) activity, which preserves microvascular integrity via the Ang-1/TIE2 signaling, playing a fundamental role in preserving lung homeostasis [15]. Additionally, our study aligns with the findings of Heikopkoski *et al*., showing that tissue levels of Ang-2 were significantly elevated in the CLP and vehicle groups compared to the sham group, with Ang-2 being recognized as a mediator of organ dysfunction during sepsis [16]. The increase in Ang-2 levels may result from releasing its stored form through toll-like receptor interactions with bacterial cell wall components or activation of NFκ-B and mitogen-activated protein kinases (MAPK)/activator protein 1 signaling pathways [17].

Furthermore, our study observed a significant reduction in tissue Ang-2 levels in the regorafenib group compared to the CLP group. To the best of our knowledge, this finding was the first to establish the effect of regorafenib on pulmonary Ang-2 in a CLP model of sepsis in mice. This finding may be due to the inhibitory effect of regorafenib on TNF-α, which is known to have a substantial relationship with Ang-2 production during sepsis [18].

Tissue levels of VEGF were significantly elevated in the CLP and vehicle groups compared to the sham group, supporting the findings of Yano *et al*., who observed that VEGF can enhance leukocyte adhesion and increase epithelial tissue sensitivity to even small amounts of TNF-α [19]. The significant increase of VEGF in CLP-induced polymicrobial sepsis may occur from the inflammatory mediators and cytokine storm characteristic of sepsis. The level of pulmonary VEGF lowered significantly in the regorafenib group compared to the CLP group, supporting the results of Liu *et al*., who found that regorafenib reduced VEGF levels in human hepatocellular carcinoma cells and decreased the secretion and expression of angiogenesis-related proteins like TNF-α and IL-1β [20]. This effect may be linked to the effect of regorafenib in inhibiting HIF-1α, a known inducer of VEGF release, which is activated by toll-like receptors during sepsis and plays a key role in the reprogramming of innate immune cells, thus potentially reducing VEGF levels and mitigating acute lung injury [21].

Our data also revealed that caspase-11 levels were notably higher in the CLP and vehicle groups compared to the sham group, which is consistent with the findings of Qiu *et al*., who showed elevated caspase-11 levels in sepsis models and demonstrated its role in mediating the inflammatory response [22]. This significant elevation in caspase-11 during sepsis can be attributed to bacterial microvesicles activating caspase-11, which in turn leads to endothelial cell pyroptosis and disrupts the endothelial barrier, facilitating pulmonary edema, the release of proinflammatory cytokines, and the recruitment of leukocytes. Furthermore, the activation of the complement system during sepsis, recognized as a key player in the ensuing inflammatory response, appears to amplify caspase-11-associated cell death [23]. In our study, lung caspase-11 significantly decreased after the administration of regorafenib compared to the CLP group. To the best of our knowledge, this study was the first to demonstrate the effect of this agent on caspase-11 in a CLP model of sepsis in mice.

Regarding tissue myeloperoxidase (MPO) levels, the regorafenib group showed a significant decrease compared to the CLP group. This supports the results of Coskun *et al*., who indicated that MPO contributes to endothelial damage and can induce the production of reactive oxygen species, leading to acute lung injury in sepsis models [24]. This may be linked to circulating LPS-inducing monocytes and neutrophils to produce MPO, which has inflammatory and oxidative effects. Neutrophils, as primary responders in sepsis, release various components, notably MPO, upon activation [24]. To our knowledge, this is the first study to demonstrate the effect of regorafenib on MPO levels in a CLP model of sepsis in mice, which may be due to its anti-inflammatory effects.

### Effect on mRNA expression of TIE2 and VE-cadherin

Our study found that mRNA expression of TIE2 and VE-cadherin was significantly lower in the CLP and vehicle groups compared to the sham group. This aligns with the findings of Ghosh *et al*., which showed a notable decrease in TIE2 expression in a sepsis mouse model promoting barrier dysfunction and contributing to inflammation and vascular leakage [25], and the observation of Huang *et al*. of reduced VE-cadherin expression in a CLP model [26]. These findings may be related to several mechanisms. First, the effect of TNFa, which is a pivotal inflammatory mediator of sepsis, acts to induce MMP14 to cleave TIE2. Second, decreased microvascular flow due to sepsis can downregulate GATA3, inhibiting TIE2 transcription [27]. As for VE-cadherin, its expression is influenced by IL1B, another crucial inflammatory mediator in sepsis, indirectly reducing VE-cadherin transcription. Furthermore, Ang-2, triggered during sepsis, can induce VE-cadherin phosphorylation, leading to a decrease in its expression [28].

In this study, we observed that regorafenib did not significantly alter TIE2 expression at the administered dose 24 hours after the induction of CLP. This result aligns with the findings of Deshors *et al*., who reported no significant reduction in TIE2 expression in glioblastoma multiforme (GBM) stem cells and tumor-derived endothelial cells treated with 1µM of regorafenib [29]. More interestingly, Abou-Elkacem *et al*. found that regorafenib significantly reduced macrophage TIE2 expression, but the effect was apparent only after 14 days of cancer cell implantation in an orthotopic murine model of colorectal cancer. This suggests that the impact of regorafenib on TIE2 expression may require an extended timeframe to manifest [5].

Although the changes in TIE2 expression were not statistically significant, these results may hold clinical relevance, as evidenced by ELISA and histopathological studies. The role of regorafenib as a tyrosine kinase inhibitor may block the binding of Ang-2 to TIE2, mitigating its adverse effects on the endothelium. Additionally, the positive influence of regorafenib on cadherin levels and the tightening of endothelial junctions offers further therapeutic value. In the current study, regorafenib significantly increased the expression of VE-cadherin in the lungs compared to the CLP group. To the best of our knowledge, this is the first instance documenting the effect of regorafenib on VE-cadherin expression in a CLP model of sepsis in mice. This finding may be due to the inhibitory effects of regorafenib on TNF-α, which is known to enhance VE-cadherin phosphorylation and reduce its expression. Moreover, the observed upregulation of VE-cadherin may also result from regorafenib's antagonistic effects on Ang-2, a known destabilizer of endothelial cell junctions [30].

### Effects on lung histopathology

The CLP and vehicle groups showed significant histopathological changes compared to the sham group. The sepsis group was characterized by the development of contraction bands, inflammatory cell infiltration, necrosis, and edema. The regorafenib group showed a significantly lower level of lung tissue injury. In addition, the spectrum of histopathological changes ranged from negligible to moderate, with a significant decrease in neutrophil infiltration, necrosis, and edema compared to the CLP and vehicle groups. To the best of our knowledge, this study was the first to demonstrate the protective effect of regorafenib on lung tissue in a mouse model of sepsis. This could be attributed to regorafenib's inhibition of the p38 mitogen-activated protein kinases and NF-κB pathways.

## CONCLUSION

Regorafenib had lung protective effects, possibly through its anti-inflammatory and antiangiogenic effects. Our study highlights the capacity of regorafenib to modulate tissue mRNA expression of TIE2 and the cadherin gene, contributing to its lung-protective mechanisms. Furthermore, it attenuated the histopathological changes during polymicrobial sepsis, reinforcing its therapeutic potential.

## References

[1] van der Poll T, Shankar-Hari M, Wiersinga WJ (2021). The immunology of sepsis. Immunity.

[2] Leligdowicz A, Richard-Greenblatt M, Wright J, Crowley VM, Kain KC (2018). Endothelial Activation: The Ang/Tie Axis in Sepsis. Front Immunol.

[3] Ettrich TJ, Seufferlein T (2018). Regorafenib. Recent Results Cancer Res.

[4] Yousif NG, Hadi NR, Al-Amran F, Zigam QA (2018). Cardioprotective effects of irbesartan in polymicrobial sepsis: The role of the p38MAPK/NF-κB signaling pathway. Herz.

[5] Abou-Elkacem L, Arns S, Brix G, Gremse F (2013). Regorafenib inhibits growth, angiogenesis, and metastasis in a highly aggressive, orthotopic colon cancer model. Mol Cancer Ther.

[6] Aziz M, Ode Y, Zhou M, Ochani M (2018). B-1a cells protect mice from sepsis-induced acute lung injury. Mol Med.

[7] Zhang Z-T, Zhang D-Y, Xie K, Wang C-J, Xu F (2021). Luteolin activates Tregs to promote IL-10 expression and alleviating caspase-11-dependent pyroptosis in sepsis-induced lung injury. Int Immunopharmacol.

[8] Tolsma V, Schwebel C, Azoulay E, Darmon M (2014). Sepsis severe or septic shock: outcome according to immune status and immunodeficiency profile. Chest.

[9] Alkharfy KM, Ahmad A, Jan BL, Raish M, Rehman MU (2022). Thymoquinone modulates the expression of sepsis-related microRNAs in a CLP model. Exp Ther Med.

[10] Hong M-K, Hu L-L, Zhang Y-X, Xu Y-L (2020). 6-Gingerol ameliorates sepsis-induced liver injury through the Nrf2 pathway. Int Immunopharmacol.

[11] Han K-M, Kang RJ, Jeon H, Lee H-J (2020). Regorafenib Regulates AD Pathology, Neuroinflammation, and Dendritic Spinogenesis in Cells and a Mouse Model of AD. Cells.

[12] Liu T, Zhang L, Joo D, Sun S-C (2017). NF-κB signaling in inflammation. Signal Transduct Target Ther.

[13] Stenzel T, Weidgang C, Wagner K, Wagner F (2016). Association of Kidney Tissue Barrier Disrupture and Renal Dysfunction in Resuscitated Murine Septic Shock. Shock.

[14] Fang Y, Li C, Shao R, Yu H (2015). Prognostic significance of the angiopoietin-2/angiopoietin-1 and angiopoietin-1/Tie-2 ratios for early sepsis in an emergency department. Crit Care.

[15] Jiang X, Tian W, Tu AB, Pasupneti S (2019). Endothelial Hypoxia-Inducible Factor-2α Is Required for the Maintenance of Airway Microvasculature. Circulation.

[16] Hepokoski M, Englert JA, Baron RM, Crotty-Alexander L (2016). Renal Angiopoietin-2 Is Increased After Injurious Mechanical Ventilation In A Murine Model Of Sepsis C49 Respiratory failure: Clinical and translational aspects of vili and lung protective MV. AM J RESP CRIT CARE.

[17] Leligdowicz A, Richard-Greenblatt M, Wright J, Crowley VM, Kain KC (2018). Endothelial Activation: The Ang/Tie Axis in Sepsis. Front Immunol.

[18] Orfanos SE, Kotanidou A, Glynos C, Athanasiou C (2007). Angiopoietin-2 is increased in severe sepsis: correlation with inflammatory mediators. Crit Care Med.

[19] Yano K, Liaw PC, Muliington JM, Shih S-C (2006). Vascular endothelial growth factor is an important determinant of sepsis morbidity and mortality. J Exp Med.

[20] Liu Y-C, Wu R-H, Wang W-S (2017). Regorafenib diminishes the expression and secretion of angiogenesis and metastasis associated proteins and inhibits cell invasion via NF-κB inactivation in SK-Hep1 cells. Oncol Lett.

[21] Fitzpatrick SF (2019). Immunometabolism and Sepsis: A Role for HIF?. Front Mol Biosci.

[22] Qiu X, Cheng X, Zhang J, Yuan C (2020). Ethyl pyruvate confers protection against endotoxemia and sepsis by inhibiting caspase-11-dependent cell pyroptosis. Int Immunopharmacol.

[23] Napier BA, Brubaker SW, Sweeney TE, Monette P (2016). Complement pathway amplifies caspase-11-dependent cell death and endotoxin-induced sepsis severity. J Exp Med.

[24] Coskun AK, Yigiter M, Oral A, Odabasoglu F (2011). The effects of montelukast on antioxidant enzymes and proinflammatory cytokines on the heart, liver, lungs, and kidneys in a rat model of cecal ligation and puncture-induced sepsis. Sci World J.

[25] Luo M, Meng J, Yan J, Shang F (2020). Role of the Nucleotide-Binding Domain-Like Receptor Protein 3 Inflammasome in the Endothelial Dysfunction of Early Sepsis. Inflammation.

[26] Huang L, Li Y, Cheng Z, Lv Z (2023). PCSK9 Promotes Endothelial Dysfunction during Sepsis via the TLR4/MyD88/NF-κB and NLRP3 Pathways. Inflammation.

[27] Tolsma V, Schwebel C, Azoulay E, Darmon M (2014). Sepsis severe or septic shock: outcome according to immune status and immunodeficiency profile. Chest.

[28] Rangasamy S, Srinivasan R, Maestas J, McGuire PG (2011). A potential role for angiopoietin 2 in the regulation of the blood-retinal barrier in diabetic retinopathy. Invest Ophthalmol Vis Sci.

[29] Deshors P, Arnauduc F, Boëlle B, Cohen-Jonathan EM (2022). Impact of regorafenib on Endothelial Transdifferentiation of Glioblastoma Stem-like Cells. Cancers (Basel).

[30] Liu L, Meng L, Zhang P, Lin H (2018). Angiotensin II inhibits the protein expression of ZO-1 in vascular endothelial cells by downregulating VE-cadherin. Mol Med Rep.

